# Pain Perceptions and Attitudes of Parents of Pediatric Age Group Patients Attending Emergency Departments in Saudi Arabia

**DOI:** 10.7759/cureus.85091

**Published:** 2025-05-30

**Authors:** Abdullah A Hammad, Raghad I Jamal Aldeen, Abeer A Alzahrani, Ittizan M Alshareef, Taif F Alkhatabi, Wed M Ismail

**Affiliations:** 1 Pediatric Emergency, King Abdullah Specialized Children Hospital, Ministry of National Guard Health Affairs, Jeddah, SAU; 2 Pediatrics, King Abdullah Specialized Children Hospital, Ministry of National Guard Health Affairs, Jeddah, SAU; 3 Pediatric Emergency, Maternity and Children Hospital, Makkah, SAU; 4 College of Medicine, King Saud Bin Abdulaziz University for Health Sciences, Jeddah, SAU

**Keywords:** emergency department, pain, parental attitudes, parental perception, pediatric age group

## Abstract

Background

Pain is the most common symptom in emergency departments and remains one of the most challenging issues for emergency care providers, especially when treating children. A family system's theoretical framework helps in understanding the development and maintenance of pain and in determining the best approaches to treating children with pain.

Objective

The objective of the study was to evaluate parental perception and attitude towards their children's acute pain and pain management, explore potential sociodemographic determinants, enhance parents' awareness and knowledge regarding pain, and provide them with practical strategies and skills to cope with pain effectively.

Methods

This prospective, descriptive, cross-sectional hospital-based study included 336 parents attending pediatric departments at the National Guard Hospital (NGHA) whose children had suffered from acute pain.

Results

Of the 336 parents/caregivers, 76.5% were female and 23.5% were male (n=257, n=79), with the most common age group being 30-39 years (n=141, 41.96%). Children showed a slight male dominance (n=188, 56%), with the majority being under three years old (n=184, 54.76%). Parents demonstrated high levels of perception regarding the potential harms of untreated pain, with 92.6% (n=311) believing it can lead to physical harm and 90.5% (n=304) believing it can lead to psychological harm. Regarding parents' attitudes, although they showed positive attitudes, Likert scale results suggested considerable uncertainty among participants. No significant associations were found between perception or attitudes about the potential harms of untreated pain and sociodemographic characteristics (p-value > 0.05 for both).

Conclusion

While pediatric pain assessment is challenging, it is crucial for parents to accurately and efficiently assess and manage their child's pain at home. Most parents demonstrate sufficient awareness of the potential harms associated with untreated pain. However, a significant proportion hold misconceptions about pharmacologic pain management due to fears of side effects.

## Introduction

The International Association for the Study of Pain defines pain as an unpleasant sensory and emotional experience associated with actual or potential tissue damage. However, this definition falls short for pediatric patients or individuals unable to express their experience. A more suitable definition for this population describes pain as a multifactorial personal experience with physiological, behavioral, emotional, developmental, and sociocultural aspects that can lead to varying perceptions of pain [1].

Pain is the most common symptom in emergency departments and remains one of the most challenging issues for emergency care providers, especially in children. It accounts for up to 80% of pediatric emergency department visits, with musculoskeletal injury being the most prevalent complaint, followed by headache, abdominal discomfort, otalgia, and sore throat [2]. Effective pain treatment in children is crucial, as inadequately treated acute pain can potentially lead to chronic pain or posttraumatic stress symptoms [3].

Parent and family factors influence the treatment of prehospital pediatric pain. A family system's theoretical framework helps in understanding the development and maintenance of pain and in determining the best approach to treating children with pain. Notably, parents of children who participated in pain-educational programs reported lower perceptions of their child's fear of pain [4,5].

A recent study conducted in Ethiopia in 2024 evaluated parental knowledge and attitudes regarding postoperative pediatric pain (POPP). The study included 102 parents who met the inclusion criteria. Results revealed that 61% of parents believed children should receive minimal pain medication to avoid side effects. Additionally, 63.4% of parents perceived that administering pain medication sparingly was the most effective pain management strategy. Notably, 26.8% of parents expressed concern that providing pain medication might encourage children to use drugs for other purposes. The study also found that parents of younger children and those from rural areas were more likely to score higher on the attention-seeking sub-score of the parental postoperative educational program (PPEP), while parents from urban areas and those who were employed demonstrated a greater awareness of pain medication side effects [6].

In a related study conducted in Botswana in 2022, Matula et al. investigated children's and parents'/guardians' perceptions (knowledge, attitudes, and beliefs) and practices regarding pediatric acute pain management. The study utilized a convenience sample of 275 parents/guardians and 42 children aged eight to 13 years, who were admitted to two Botswana tertiary hospitals between November 2018 and February 2019. Analysis of the survey data revealed that all subscales, except for parents'/guardians' pain interference (p=0.96), were statistically significant (p=0.00). These findings indicated adequate knowledge, positive attitudes, and high-pain intensity perceptions among both parents/guardians and children [7].

A study conducted in Saudi Arabia in 2021 assessed mothers' attitudes toward their children's pain management, focusing on non-pharmacological approaches. The majority of participating mothers (62.6%, 250) relied primarily on rest to alleviate their child's pain [8].

Effective pain management in pediatric patients requires comprehensive educational strategies targeting children, caregivers, and emergency department personnel. Caregiver refusal of analgesia can impede adequate pain management in children; therefore, educational interventions are crucial to dispel misconceptions and promote acceptance of appropriate analgesic use [9,10]. Conversely, parental misuse or overuse of analgesics can adversely affect children's health. Parental influence on children's pain experiences is significant, with their own past experiences correlating with their perception of their child's pain. Parents may harbor concerns about analgesic side effects, potential for addiction, and may believe in minimizing pain medication administration [11].

Despite extensive educational efforts across the continuum of care, particularly in pre-hospital settings advocating for early pediatric pain assessment and management, the recognition, assessment, and treatment of pre-hospital pain remain suboptimal. This presents an opportunity for quality improvement initiatives [12]. Consequently, there is a need for research exploring parental, especially maternal, experiences with pain management before and during their child's emergency department visit.

This study aimed to address the research gap in understanding parental perceptions and attitudes toward acute pain and pain management in children within the Saudi Arabian context. To the best of our knowledge, limited studies have been conducted in this region to assess this critical aspect of pediatric care.

Therefore, the objective of our study was to evaluate parental perceptions and attitudes toward their children's acute pain and pain management, as well as to explore potential sociodemographic determinants. Additionally, we sought to enhance parents' awareness and knowledge regarding pain and provide them with practical strategies and skills to effectively cope with it.

## Materials and methods

A descriptive, cross-sectional study was conducted in the emergency department of the National Guard Hospital (NGHA) in Jeddah, Saudi Arabia. The study period extended from July 2023 to January 2024, encompassing a six-month data collection phase. The study population consisted of parents or primary caregivers accompanying children under 13 years of age who presented to the pediatric emergency department with acute pain. Inclusion criteria encompassed parents or primary caregivers seeking medical attention for their children within the specified age range. Exclusion criteria were as follows: individuals with unstable mental status, those experiencing significant psychological distress in the emergency department, parents or caregivers who declined to provide informed consent, and non-Arabic speaking participants. 

The number of participants required to estimate the level of knowledge was calculated using this equation:

$n=\frac{P \left( 1-P \right) z^2}{d^{2}}$

In light of the absence of studies conducted in Saudi Arabia specifically assessing parental perceptions and attitudes toward their children's pain, findings from a related study in Riyadh were referenced. This study evaluated parents' perceptions and practices concerning the home management of fever and revealed that approximately one-third of participants (33.3%) reported difficulties in managing their febrile children [13]. These results served as a benchmark for estimating parental perceptions in the current study when calculating the required sample size. Accordingly, with a confidence level of 95% and an allowable margin of error of 0.05, the sample size was calculated as follows:

$n=\frac{0.33\left( 1-0.33 \right) 1.96^{2}}{0.05^{2}}$ = 336 participants

This study employed a systematic random sampling technique to select participants from hospital records, ensuring an unbiased representation of parents and caregivers. Data collection was conducted through structured interviews with these individuals in the pediatric emergency department during their visit. 

Participants completed a self-administered questionnaire designed for data collection on the study variables. The instrument comprised three sections (see Table 5 in Appendices): Section A focused on the demographic characteristics of the parents; Section B addressed child-related characteristics; and Section C explored perceptions and attitudes toward pain and pain management. The questionnaire contained a validated scale to assess medication attitudes and included additional questions regarding parental perceptions. To investigate how parents perceived the impact of untreated pain in children, they were asked to indicate their agreement or disagreement with two specific questions: "Do you believe that untreated pain can lead to physical harm?" and "Do you believe that untreated pain can lead to psychological harm?" Parents were expected to provide a simple "yes" or "no" response to each question.

For the assessment of parents’ attitudes, the Medication Attitude Questionnaire (MAQ) was adopted from a previous study to explore attitudes regarding the use of pain medication for the treatment of children's pain [14]. Permission was sought from the authors to utilize their questionnaire. The MAQ included 15 questions, with each item rated on a seven-point Likert scale ranging from "strongly disagree" (1) to "strongly agree" (7). Parents were instructed to consider analgesia as any medication recommended for a specific event or over-the-counter analgesia at any time. The overall scale's internal consistency (Cronbach's alpha) was reported to range between 0.68 and 0.73, while the internal consistency coefficients for the four subscales ranged from 0.63 to 0.75.

Following ethical approval and participant consent, data were collected and systematically organized using Microsoft Excel (Microsoft Corp., Redmond, United States) to create a comprehensive database. Statistical analysis was conducted using IBM SPSS Statistics Version 26 (IBM Corp., Armonk, United States). Descriptive statistics were employed to summarize the data, with frequencies and percentages calculated for categorical variables, and means, medians, standard deviations, and ranges computed for continuous variables.

To assess associations between sociodemographic characteristics and parental perceptions, chi-squared tests were performed. Due to the non-normal distribution of the data, non-parametric tests were utilized to evaluate the relationship between sociodemographic factors and parental attitudes. The Kruskal-Wallis test was employed for comparisons involving three or more independent groups, while the Mann-Whitney U test was applied for two-group comparisons. Statistical significance was set at p<0.05 for all analyses.

Ethical approval was sought before starting the study. The objectives and benefits of the study were explained to the participants. Informed consent was taken from each participant before collecting the data. Confidentiality and privacy of participants were maintained. The participants had the right to withdraw consent at any time without any consequences.

## Results

Demographic analysis of the study participants revealed several significant findings. Female participants constituted a majority (76.5%, n=257) of the sample, indicating strong maternal representation. The predominant age group was 30-39 years (41.96%, n=141), suggesting a prevalence of younger parents. Marital status data indicated that the vast majority of participants were married (95.5%, n=321). Regarding family composition, 48.51% (n=163) reported having fewer than three children, while 43.75% (n=147) had three to five children. Educational attainment analysis showed that 59.8% (n=201) held university degrees. In terms of employment status, 42.9% (n=144) were unemployed, with only a small fraction (2.4%, n=8) identifying as students. The prevalence of chronic illness was relatively low, affecting 10.7% (n=36) of parents and 18.2% (n=61) of children. Child demographic data revealed a slight male predominance (56%, n=188), with the majority (54.76%, n=184) being under three years of age. Comprehensive demographic details are presented in Table 1.

**Table 1:** Sociodemographic data of participants (n=336) The table shows the frequencies and percentages of each sociodemographic variable.

Sociodemographic	No.	%
Parent's gender		
Male	79	23.5
Female	257	76.5
Parent's age		
20 to 29 years	87	25.89
30 to 39 years	141	41.96
40 to 49 years	91	27.08
50 to 60 years	17	5.06
Marital status		
Married	321	95.5
Divorced/widowed	15	4.5
Number of children		
Less than 3 children	163	48.51
From 3 to 5 children	147	43.75
More than 5 children	26	7.74
Education level		
Primary education	14	4.2
Secondary education	85	25.3
University level	201	59.8
Postgraduate level	36	10.7
Occupation		
Government employee	128	38.1
Private sector employee	56	16.7
Student	8	2.4
Unemployed	144	42.9
Do you have a chronic illness?		
Yes	36	10.7
No	300	89.3
Does your child have a chronic illness?		
Yes	61	18.2
No	275	89.3
Child's gender		
Male	188	56
Female	148	44
Child's age		
Less than 3 years	184	54.76
4 to 6 years	58	17.26
7 to 10 years	60	17.86
More than 10 years	34	10.12

**Figure 1:**
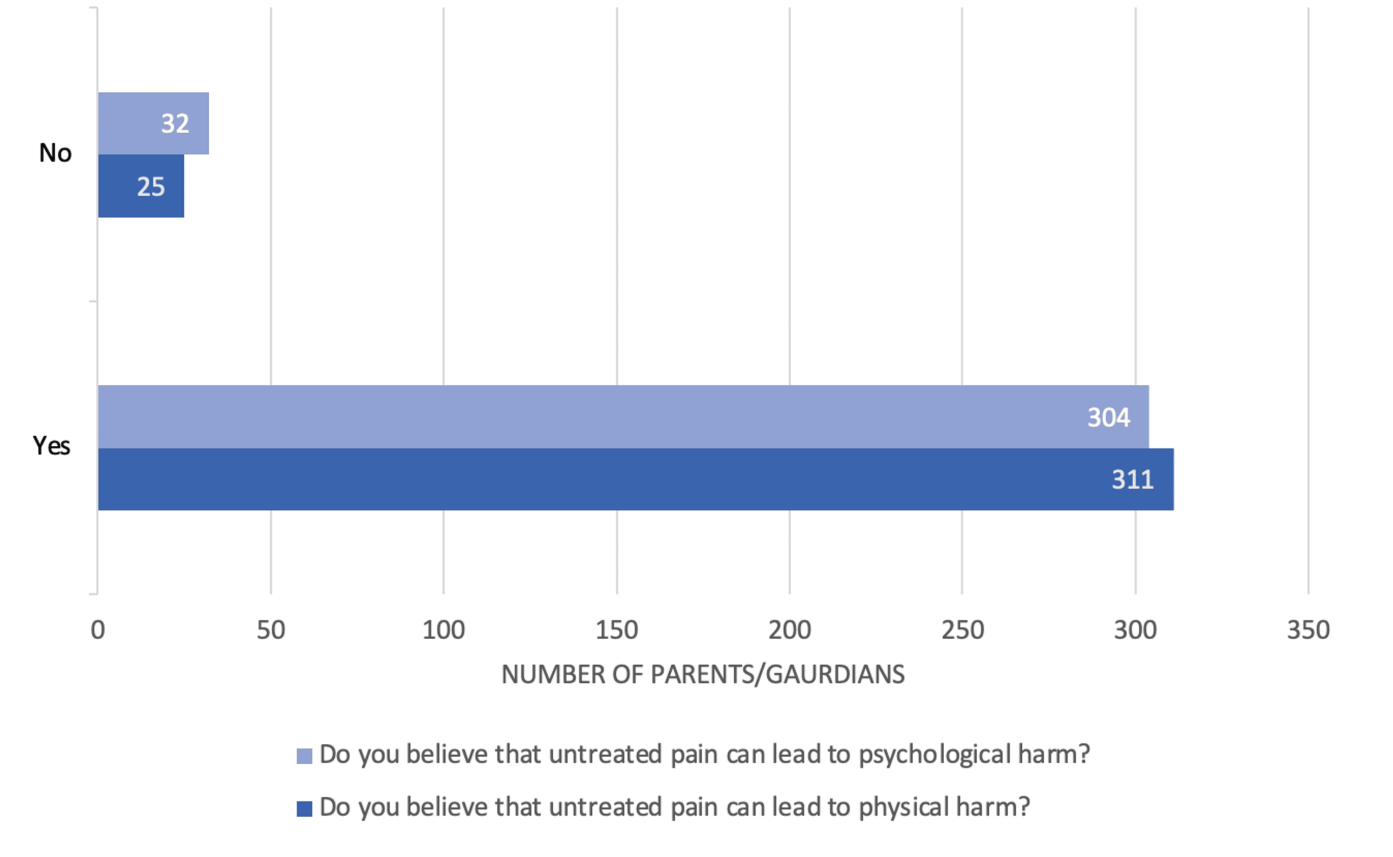
Parental perception regarding the effects of untreated pain

The analysis of parental beliefs about the consequences of untreated pain revealed noteworthy statistical findings. A substantial majority of parents (92.6%, n=311) believed that untreated pain could lead to physical harm, while only 7.4% (n=25) disagreed. In terms of psychological consequences, 90.5% (n=304) of parents expressed the belief that untreated pain could result in psychological harm, with 9.5% (n=32) not sharing this view. The slightly lower percentage of agreement regarding psychological harm, coupled with a higher proportion of differing opinions (9.5%), suggests a marginally greater uncertainty about the psychological impacts of untreated pain. The frequency distribution of responses, based on a sample of 336 participants, is illustrated in Figure 1, demonstrating a strong awareness among parents regarding the potential harms associated with untreated pain in children.

**Figure 2:**
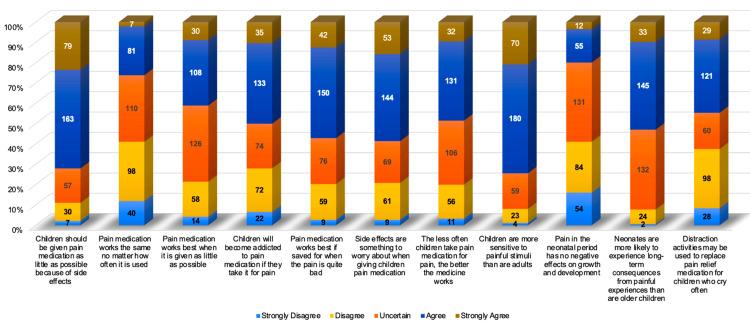
Parental attitudes toward pain management based on the Likert scale

Table 2 summarizes the statistical analysis of parents' attitudes toward pain medication for children. A majority of parents (72%, n=242) agreed that pain medication should be administered sparingly due to concerns about potential side effects. Notably, 32.7% (n=110) expressed uncertainty about whether pain medication works the same regardless of use frequency. Many parents were also uncertain about the effectiveness of infrequent medication administration. Concerns about addiction did not resonate strongly, as many remained unsure about dependency risks. In contrast, 57.1% (n=192) agreed that pain medication should be reserved for severe discomfort. Additionally, 58.7% (n=197) acknowledged concerns about side effects when treating their children. While 48.5% (n=163) agreed that less frequent use of medication enhances efficacy, 74.4% (n=250) recognized that children are more sensitive to pain than adults. However, 41.1% (n=138) were unaware of the long-term effects of neonatal pain on development. Agreement from 53% (n=178) that neonates may face severe long-term consequences underscores their vulnerability. Mixed responses on distraction as an alternative to medication (44.6% agree, 37.5% disagree) indicated uncertainty (Figure 2).

**Table 2:** Frequency distribution of parents' attitudes toward pain medication for children (n=336) The table shows the frequency distribution for the parents' attitudes section. The attitudes were measured using a five-point Likert scale, which ranges from "strongly disagree" to "strongly agree." This scale allows respondents to express the degree of their agreement or disagreement with various statements regarding pain management practices.

Assessment of Parents' Attitudes	Strongly Disagree		Disagree		Uncertain		Agree		Strongly Agree		Mean ± SD	5-Point Likert Scale
	n	%	n	%	n	%	n	%	n	%		
Children should be given pain medication as little as possible because of the side effects	7	2.1%	30	8.9%	57	17%	163	48.5%	79	23.5%	3.82 ± 0.96	Agree
Pain medication works the same no matter how often it is used	40	11.9%	98	29.2%	110	32.7%	81	24.1%	7	2.1%	2.75 ± 1.02	Uncertain
Pain medication works best when it is given as little as possible	14	4.2%	58	17.3%	126	37.5%	108	32.1%	30	8.9%	3.24 ± 0.98	Uncertain
Children will become addicted to pain medication if they take it for pain	22	6.5%	72	21.4%	74	22%	133	39.6%	35	10.4%	3.26 ± 1.11	Uncertain
Pain medication works best if saved for when the pain is quite bad	9	2.7%	59	17.6%	76	22.6%	150	44.6%	42	12.5%	3.47 ± 1.01	Agree
Side effects are something to worry about when giving children pain medication	9	2.7%	61	18.2%	69	20.5%	144	42.9%	53	15.8%	3.51 ± 1.05	Agree
The less often children take pain medication for pain, the better the medicine works	11	3.3%	56	16.7%	106	31.5%	131	39%	32	9.5%	3.35 ± 0.97	Uncertain
Children are more sensitive to painful stimuli than are adults	4	1.2%	23	6.8%	59	17.6%	180	53.6%	70	20.8%	3.86 ± 0.86	Agree
Pain in the neonatal period has no negative effects on growth and development	54	16.1%	84	25%	131	39%	55	16.4%	12	3.6%	2.66 ± 1.04	Uncertain
Neonates are more likely to experience long-term consequences from painful experiences than are older children	2	0.6%	24	7.1%	132	39.3%	145	43.2%	33	9.8%	3.54 ± 0.79	Agree
Distraction activities may be used to replace pain relief medication for children who cry often	28	8.3%	98	29.2%	60	17.9%	121	36%	29	8.6%	3.07 ± 1.15	Uncertain
Overall	200	5.41%	663	17.94%	1000	27.06%	1411	38.18%	422	11.42%	3.32 ± 0.99	Uncertain

Analysis of factors influencing parental perceptions of harm from untreated pain revealed no statistically significant associations with various demographic variables (Table 3). Parental gender showed no significant relationship with perceived physical harm (p=0.587) or psychological harm (p=0.191). Similarly, parental age demonstrated no significant association with either perceptions of physical harm (p=0.646) or psychological harm (p=0.231). Marital status also exhibited no significant correlation with perceived physical harm (p=0.291) or psychological harm (p=0.215). Analysis of the number of children revealed no significant association with perceptions of physical harm (p=0.336) or psychological harm (p=0.248). Similarly, educational attainment showed no significant correlation with perceptions of physical harm (p=0.155) or psychological harm (p=0.813). Occupational status also demonstrated no significant relationship with perceptions of physical harm (p=0.264) or psychological harm (p=0.687). Additionally, the presence of chronic illness in either parents or children did not significantly influence perceptions of harm, as evidenced by the non-significant p-values across both groups.

**Table 3:** Inferential analysis of parents' perception in relation to sociodemographic variables (n=336) The table shows the results of the inferential analysis for the parents' perception section. This section includes two yes or no questions, which are considered categorical variables. The chi-squared test was performed to measure the association between the sociodemographic variables and parents' perception. Df: Degree of freedom; EF: Effect size; *: p<0.05 (significant)

Variables		Untreated Pain Can Lead to Physical Harm		Untreated Pain Can Lead to Psychological Harm		Physical Harm Test Statistics _(EF,Df)_	Psychological Harm Test Statistics _(EF,Df)_	p-value (Psychological Harm)	p-value (Psychological Harm)
		Yes	No	Yes	No				
		n (%)	n (%)	n (%)	n (%)				
Parent's gender	Male	74 (22.02)	5 (1.49)	70 (20.83)	9 (2.68)	0.956_(1,0.052)_	1.178_(1,0.052)_	0.587	0.191
	Female	237 (70.54)	20 (5.95)	234 (69.64)	23 (6.85)				
Parent's age	20 to 29 years	80 (23.81)	7 (2.08)	79 (23.51)	8 (2.38)	1.659_(3,0.040)_	4.303_(3,0.064)_	0.646	0.231
	30 to 39 years	129 (38.39)	12 (3.57)	129 (38.39)	12 (3.57)				
	40 to 49 years	85 (25.3)	6 (1.79)	79 (23.51)	12 (3.57)				
	50 to 60 years	17 (5.06)	0 (0)	17 (5.06)	0 (0)				
Marital status	Married	296 (88.1)	25 (7.44)	289 (86.01)	32 (9.52)	1.317_(1,0.069)_	1.653_(1,0.069)_	0.291	0.215
	Divorced/widowed	15 (4.46)	0 (0)	15 (4.46)	0 (0)				
Number of children	Less than 3 children	153 (45.54)	10 (2.98)	149 (44.35)	14 (4.17)	2.181_(2,0.054)_	2.790_(2,0.053)_	0.336	0.248
	From 3 to 5 children	133 (39.58)	14 (4.17)	133 (39.58)	14 (4.17)				
	More than 5 children	25 (7.44)	1 (0.3)	22 (6.55)	4 (1.19)				
Education level	Primary education	12 (3.57)	2 (0.6)	13 (3.87)	1 (0.3)	5.238_(3,0.072)_	0.950_(3,0.031)_	0.155	0.813
	Secondary education	76 (22.62)	9 (2.68)	75 (22.32)	10 (2.98)				
	University level	187 (55.65)	14 (4.17)	182 (54.17)	19 (5.65)				
	Postgraduate level	36 (10.71)	0 (0)	34 (10.12)	2 (0.6)				
Occupation	Government employee	123 (36.61)	5 (1.49)	116 (34.52)	12 (3.57)	4.144_(3,0.064)_	1.438_(3,0.036)_	0.264	0.687
	Private sector employee	50 (14.88)	6 (1.79)	52 (15.48)	4 (1.19)				
	Student	8 (2.38)	0 (0)	8 (2.38)	0 (0)				
	Unemployed	130 (38.69)	14 (4.17)	128 (38.1)	16 (4.76)				
Do you have a chronic illness?	Yes	32 (9.52)	4 (1.19)	32 (9.52)	4 (1.19)	1.943_(1,0.076)_	0.768_(1,0.043)_	0.143	0.267
	No	279 (83.04)	21 (6.25)	272 (80.95)	28 (8.33)				
Does your child have a chronic illness?	Yes	55 (16.37)	6 (1.79)	53 (15.77)	8 (2.38)	0.523_(1,0.043)_	1.230_(1,0.061)_	0.31	0.19
	No	256 (76.19)	19 (5.65)	251 (74.7)	24 (7.14)				
Child's gender	Male	171 (50.89)	17 (5.06)	166 (49.4)	22 (6.55)	1.017_(1,0.055)_	1.343_(1,0.059)_	0.212	0.166
	Female	140 (41.67)	8 (2.38)	138 (41.07)	10 (2.98)				
Child's age	Less than 3 years	169 (50.3)	15 (4.46)	166 (49.4)	18 (5.36)	2.647_(3,0.051)_	0.483_(3,0.022)_	0.481	0.923
	4 to 6 years	55 (16.37)	3 (0.89)	51 (15.18)	7 (2.08)				
	7 to 10 years	55 (16.37)	5 (1.49)	55 (16.37)	5 (1.49)				
	More than 10 years	32 (9.52)	2 (0.6)	32 (9.52)	2 (0.6)				

Analysis of child-related factors revealed no statistically significant associations between gender or age and parental perceptions of harm from untreated pain. For gender, p-values were 0.212 and 0.166 for physical and psychological harm, respectively. Similarly, for age, p-values were 0.481 and 0.923.

Table 4 presents the inferential analysis results for parental attitudes. No statistically significant differences were observed across various sociodemographic variables. Parental gender (p=0.937), age (p=0.950), marital status (p=0.404), number of children (p=0.313), educational attainment (p=0.412), and occupation (p=0.995) all demonstrated p-values exceeding the conventional threshold of statistical significance (p<0.05). These findings suggest that parental attitudes toward children's pain management remained consistent across diverse demographic subgroups within our study population. Analysis of chronic illness status revealed no significant differences in parental attitudes, with p-values of 0.483 for parents and 0.191 for children. Similarly, child-specific factors such as gender (p=0.127) and age (p=0.144) demonstrated no statistically significant impact on parental attitudes. These findings collectively suggest that demographic characteristics do not exert a significant influence on parental attitudes toward children's pain management in our study population.

**Table 4:** Inferential analysis of parents' attitudes in relation to sociodemographic variables (n=336) The table shows the results of the inferential analysis for the parents' attitudes section, which consisted of 11 Likert scale questions. A new continuous variable, termed "parents' attitudes," was measured by calculating the sum of each participant's answers to the 11 questions. a: Kruskal-Wallis test; b: Mann-Whitney U test; *: p<0.05 (significant)

Variables		Mean ± SD	p-value
Total		36.54 ± 4.94	N/A
Parent's gender	Male	36.68 ± 36.51	0.937_b_
	Female	5.22 ± 4.87	
Parent's age	20 to 29 years	36.53 ± 4.98	0.950_a_
	30 to 39 years	36.5 ± 4.99	
	40 to 49 years	36.56 ± 4.66	
	50 to 60 years	36.94 ± 6.22	
Marital status	Married	36.5 ± 4.95	0.404_b_
	Divorced/widowed	37.53 ± 4.98	
Number of children	Less than 3 children	36.33 ± 5.19	0.313_a_
	From 3 to 5 children	36.95 ± 4.72	
	More than 5 children	35.62 ± 4.53	
Education level	Primary education	37.36 ± 5.12	0.412_a_
	Secondary education	36.62 ± 5	
	University level	36.74 ± 4.83	
	Postgraduate level	34.97 ± 5.29	
Occupation	Government employee	36.52 ± 5.04	0.995_a_
	Private sector employee	36.95 ± 5.1	
	Student	36.13 ± 3.56	
	Unemployed	36.44 ± 4.89	
Do you have a chronic illness?	Yes	36.89 ± 6.26	0.483_b_
	No	36.51 ± 4.77	
Does your child have a chronic illness?	Yes	37.26 ± 4.32	0.191_b_
	No	36.39 ± 5.07	
Child's gender	Male	36.21 ± 4.92	0.127_b_
	Female	36.97 ± 4.96	
Child's age	Less than 3 years	36.42 ± 5.04	0.144_a_
	4 to 6 years	35.91 ± 4.69	
	7 to 10 years	36.87 ± 4.42	
	More than 10 years	37.76 ± 5.66	

## Discussion

This study aimed to evaluate parental perceptions and attitudes toward their children's acute pain and pain management, as well as explore potential sociodemographic determinants. Additionally, it sought to enhance parents' awareness and knowledge regarding pain and provide them with practical strategies and skills to effectively manage their children’s pain.

Recent literature has shifted focus toward parents, particularly mothers, managing their children's pain at home. This shift can be attributed to the growing recognition that parents address a significant portion of children's pain experiences in home settings. However, despite parents being the primary caregivers for their children, studies have noted limited knowledge, attitudes, and practices regarding both pharmacological and non-pharmacological pain management strategies among parents [6].

While no study has been conducted in Saudi Arabia on this exact topic, several studies in the region have explored related objectives. Furthermore, numerous published studies from other regions have addressed similar topics, providing a broader context for the present investigation.

For example, the study of Alghadeer et al. in Saudi Arabia in 2021 found that the non-pharmacological methods used by mothers at home for the management of their children's pain were as follows: letting them take rest or sleep (n=250, 62.6%), feeding them with fluids (n=228, 57.1%), applying cold packs (n=161, 40.4%), providing massage therapy (n=147, 36.8%), using warm packs (n=141, 35.3%), and taking them to play (n=119, 29.8%). These findings show that the mothers had poor attitudes regarding management of their children's pain, which is dissimilar to the results of our study, which revealed that parents demonstrated better attitudes; however, there was a considerable level of uncertainty among participants. The only common attitude between the two studies is using distraction as a pain relief method, in which the current study showed that 44.6% (n=150) believed that distraction activities may be used to replace pain relief medication for children who cry often [8]. On the contrary, a Korean study reported that using distraction for children’s pain relief was not among the frequently used methods of parental acute pain management, unlike emotional support approaches such as consoling and parental engagement [15]. The preferred method of non-pharmacological pain management at home differed from one geographical region to another, highlighting the influence of culture on children's pain management.

A study in Turkey assessing pain relief interventions performed prior to pediatric emergency department visits involved 425 pediatric patients with a mean age of 8.16 ± 4.03. Gender distribution among patients was almost equal, with 50.8% female and 49.2% male. In this study, most parents intervened with their child’s pain before visiting the emergency department. The most common intervention used by parents was the administration of pharmacological treatment (81%), which could be explained by the wide availability and rapid action of analgesics and paracetamol in managing acute pain. A significant relationship was found between the classification of pain and parental employment status when administering medications. Moreover, a statistically significant association between employed parents and initial parental involvement was established [16]. The findings of this study oppose the findings in our study, as parents in our study mostly believed that children should be given pain medication cautiously due to possible side effects.

The main reason for parents' unwillingness to allow their children to take drugs could be due to their inadequate knowledge about pain and analgesics, as well as the benefits and safety of analgesics. Even the mothers' approaches to pain relief and their attitudes toward their children's postoperative pain were found to be unsatisfactory [17]. However, a study reported a significant association between guardians’ level of knowledge, experience, and attitude toward their children’s postoperative pain and satisfaction [18].

Similar results were found in the literature, particularly in Finland, as Palomaa et al. stated that parents reported their children experienced moderate (36%) to severe pain (42%) during hospitalization. The most intense pain experienced by the children was associated with needle-related procedures (41%). A large proportion of parents (83%) were involved in their child's pain assessment. Parents were satisfied with their child's pain assessment but perceived some shortcomings. Parents hoped that a variety of methods would be used to assess their child's pain and that the parents' and child's views on pain would be considered. Thus, the authors demonstrated that the majority of parents had good perception toward their children's pain, which aligns with the findings of our study, in which high levels of perception were noticed among parents about the potential harms associated with untreated pain, with the majority (92.6%) believing that untreated pain can lead to physical harm, and 90.5% of parents believing that untreated pain can lead to psychological harm. It is vital that both the child and parents are included in shared decision-making about pain assessment and treatment and that they have opportunities to ask questions. Guidance should be accessible for parents about the use of pain assessment scales [19].

A comparative analysis was conducted between the findings of the present study and a recent study by Desalegn et al. (2024) in Ethiopia [6]. The Ethiopian study, with a smaller sample size of 102 parents compared to our 336, may have limitations in terms of generalizability. Regarding parental attitudes toward pain medication, both studies revealed similar concerns about side effects, with 61% of Ethiopian parents and 72% of Saudi parents believing children should receive minimal pain medication due to potential side effects.

Notably, there were differences in perceptions of pain medication risks. In Ethiopia, 26.8% of parents believed that administering pain medication might lead to future drug misuse, while in Saudi Arabia, 50% of parents expressed concerns about potential addiction. Additionally, 63.4% of Ethiopian parents considered minimal pain medication as the most effective pain management strategy, compared to 41% in Saudi Arabia [6]. Indistinguishable findings were also reported in a recent cross-sectional study that evaluated the level of understanding and awareness of paracetamol use in children among Palestinian caregivers. About 46% of participants were aware of the serious complications of paracetamol overuse in children. However, almost all parents (95.5%) lacked adequate knowledge regarding paracetamol dosage [20].

The Ethiopian study reported associations between sociodemographic factors and parental perceptions, with parents of younger children and those from rural areas scoring higher on attention-seeking behaviors of the PPEP, while urban and employed parents showed greater concern about medication side effects. In contrast, our study found no significant associations between sociodemographic characteristics and perceptions of untreated pain risks (p-value > 0.05) [6]. Another study in Italy measuring parental obedience to international regulations of child pain management showed different results, where parents with a college degree scored significantly higher grades than those with lower educational levels. However, comparable to the Ethiopian study, parents with older children aged 6-14 years scored lower on the obtained questionnaire regarding adherence to acute pain and fever management guidelines in children [21]. These findings highlight the need for further research to understand the cultural and socioeconomic factors influencing parental attitudes toward pediatric pain management across different populations [6].

A study conducted by Matula et al. (2022) in Botswana examined pain perceptions among 275 parents/guardians and 42 children aged 8-13 years admitted to two tertiary hospitals between November 2018 and February 2019 [7]. The study revealed that 47% (n=129) of parents/guardians reported moderate to severe pain in their children, while 38% (n=16) of children self-reported moderate to severe pain at the time of the survey. The mean scores for the children's modified American Pain Society Patient Outcome Questionnaire-Revised (cm-APS-POQ-R) were 113 (SD=33), and for parents/guardians (m-APS-POQ-R) were 123 (SD=26). All subscales, except for parents'/guardians' pain interference (p=0.96), showed statistical significance (p<0.001), indicating adequate knowledge, positive attitudes, and high pain intensity for both parents/guardians and children [7].

In contrast, our study in Saudi Arabia demonstrated that parents exhibited positive attitudes toward pain management. However, the Likert scale results suggested considerable uncertainty among participants. Moreover, no significant associations were found between perceptions and attitudes about potential harms of untreated pain among parents and sociodemographic characteristics (p>0.05). These findings highlight the need for further research to understand cultural and contextual factors influencing parental attitudes toward pediatric pain management across different populations [7].

A study by Lee et al. (2020) in the United States reported findings that contradict our results [22]. Their study found that higher daily pain intensity was associated with increased oxycodone administration, but not acetaminophen or ibuprofen use. Additionally, positive parental attitudes toward complementary and alternative medicine (CAM) were associated with reduced oxycodone use, independent of the child's daily pain intensity and average postoperative pain. Both parental CAM attitudes and the child's daily pain intensity were independently associated with parental decisions to administer opioids [22].

In contrast, our study revealed that 72% of parents agreed that children should receive minimal pain medication due to concerns about side effects. This finding reflects a strong apprehension regarding the potential adverse effects of pain medication. Furthermore, we found no significant association between parental attitudes about the potential harms of untreated pain and sociodemographic characteristics (p>0.05) [22].

As proven by many studies worldwide, female parents serve as the primary caregivers within their families [23,24]. They also account for 73.8% of informal caregivers/social workers among caregivers in Spain [25]. Likewise, most of our study participants were female (76.5%). This could be explained by the emotional and caretaking nature of most female individuals as well as the cultural norms in most countries, especially in the Arab culture. Rothenberg and others studied the socioemotional caregiving practices and early childhood development among 51 countries of low-middle income. They concluded that mothers were mostly involved in socioemotional caregiving practices when compared to fathers and informal caregivers. It was also inferred that the more engaged mothers were in taking care of their children, the better they progress developmentally [26]. Therefore, educational programs regarding children's pain management and attitudes should highly encourage and target female parents/mothers to achieve greater benefits. 

In summary, it was noticed that some of the results of the studies found in the literature aligned with those found in the present study in Saudi Arabia, while others were dissimilar. However, the findings of this study provide new evidence in the region, supporting the evidence found in other regions and bridging the gap in the literature. Furthermore, the variety of findings among study results could be due to cultural and societal differences across different regions, levels of education in each country, and the differences in the objectives of each study. However, one common aspect in all studies is that the knowledge, perception, and attitudes about the potential harms associated with untreated pain need more attention and effort.

Strengths and limitations

This study is the first in Saudi Arabia to explore parental perceptions and attitudes toward children’s acute pain and pain management, addressing a critical clinical issue. It provides valuable insights into factors influencing parental knowledge and practices in managing pediatric pain at home, which could potentially reduce unnecessary emergency room (ER) visits by improving home-based pain management.

However, the study is restricted by its single-site cross-sectional design, as causal inferences are limited. Further multicenter research is needed to enhance the generalizability of results. The extent to which participants were responsible for managing their child’s pain at home is unclear, and although the questionnaires were translated into Arabic, cultural relevance was not specifically addressed. 

Overall, while the study offers important insights, future research is needed to further explore these factors and their potential impact on reducing ER load across diverse settings.

## Conclusions

In conclusion, pediatric pain assessment presents a significant challenge, yet it is imperative for parents to adeptly and efficiently evaluate and manage their child's pain at home. The study indicates that most parents who visited the emergency department of NGHA in Jeddah with their children possessed an adequate understanding of the risks associated with untreated pain. However, there were some parents who still harbored misconceptions about postoperative pharmacological pain management, primarily due to fears of potential side effects. Given the critical role parents play and the existing gap in research on their attitudes and perceptions toward pain assessment and management, this study aimed to elevate parental awareness and knowledge about pain. It also sought to equip parents with practical strategies and skills to effectively address and mitigate pain.

## Appendices

**Table 5:** Data collection sheet

Section A: Sociodemographic Data									
Parent's gender:									
Male					Female				
Parent's age:									
Marital status:									
Single		Married			Divorced			Widow	
Number of children:									
Educational level:									
Primary education		Secondary education			University level			Postgraduate level	
What is your occupation?									
Government employee		Private sector employee			Student			Unemployed	
Do you have any chronic illnesses?									
Yes					No				
Does your child have any chronic illnesses?									
Yes					No				
Child's gender:									
Male					Female				
Child's age in months:									
Section B: Parents’ Perception									
Do you believe that untreated pain can lead to physical harm?									
Yes					No				
Do you believe that untreated pain can lead to psychological harm?									
Yes					No				
Section C: Parents’ Attitudes									
Children should be given pain medication as little as possible because of the side effects:									
Strongly disagree	Disagree		Slightly disagree	Uncertain		Slightly agree	Agree		Strongly Agree
Pain medication works the same no matter how often it is used:									
Strongly disagree	Disagree		Slightly disagree	Uncertain		Slightly agree	Agree		Strongly Agree
Pain medication works best when it is given as little as possible:									
Strongly disagree	Disagree		Slightly disagree	Uncertain		Slightly agree	Agree		Strongly Agree
Pain medication has many side effects:									
Strongly disagree	Disagree		Slightly disagree	Uncertain		Slightly agree	Agree		Strongly Agree
Children will become addicted to pain medication if they take it for pain:									
Strongly disagree	Disagree		Slightly disagree	Uncertain		Slightly agree	Agree		Strongly Agree
There is little need to worry about side effects from pain medication:									
Strongly disagree	Disagree		Slightly disagree	Uncertain		Slightly agree	Agree		Strongly Agree
It is unlikely a child will become addicted to pain medication if taken for pain:									
Strongly disagree	Disagree		Slightly disagree	Uncertain		Slightly agree	Agree		Strongly Agree
Pain medication is addictive:									
Strongly disagree	Disagree		Slightly disagree	Uncertain		Slightly agree	Agree		Strongly Agree
Pain medication works best if saved for when the pain is quite bad:									
Strongly disagree	Disagree		Slightly disagree	Uncertain		Slightly agree	Agree		Strongly Agree
Side effects are something to worry about when giving children pain medication:									
Strongly disagree	Disagree		Slightly disagree	Uncertain		Slightly agree	Agree		Strongly Agree
The less often children take pain medication for pain, the better the medicine works:									
Strongly disagree	Disagree		Slightly disagree	Uncertain		Slightly agree	Agree		Strongly Agree
Children are more sensitive to painful stimuli than are adults:									
Strongly disagree	Disagree		Slightly disagree	Uncertain		Slightly agree	Agree		Strongly Agree
Pain in the neonatal period has no negative effects on growth and development:									
Strongly disagree	Disagree		Slightly disagree	Uncertain		Slightly agree	Agree		Strongly Agree
Neonates are more likely to experience long-term consequences from painful experiences than are older children:									
Strongly disagree	Disagree		Slightly disagree	Uncertain		Slightly agree	Agree		Strongly Agree
Distraction activities may be used to replace pain relief medication for children who cry often:									
Strongly disagree	Disagree		Slightly disagree	Uncertain		Slightly agree	Agree		Strongly Agree
